# Photoacoustic imaging of acupoint specificity and meridian transmission at Shousanli (LI10): a blood oxygen metabolism study

**DOI:** 10.3389/fphys.2026.1911653

**Published:** 2026-08-21

**Authors:** Cheng Zeng, Yameng Liu, Linping Pian, Jingjing Zhao, Linying Wang, Liping Pei, Jiawei Liu, Xueyan Gao, Danke Ma, Jing Gao

**Affiliations:** 1 Henan Univ Tradit Chinese Med, Dept Ultrasound Med, Zhengzhou, China; 2 Henan Univ Chinese Med, Affiliated Hosp 1, Dept Ultrasound Med, Zhengzhou, China

**Keywords:** acupoint specificity, acupuncture, blood oxygen metabolism, meridian transmission, photoacoustic imaging, Shousanli

## Abstract

**Objective:**

To examine how acupuncture at Shousanli (LI10) influences blood oxygen metabolism in tissues beneath acupoints along the Hand-Yangming Large Intestine Meridian, as well as at non-meridian sites, with the aim of validating acupoint specificity and meridian transmission.

**Methods:**

60 healthy volunteers were randomized to acupoint (n=30, LI10) or non-meridian (n=30, 2 cm medial to LI10) groups. Photoacoustic imaging assessed oxygen saturation (SO_2_) at LI10, LI11, LI8, LI4, and corresponding non-meridian sites at baseline, post-deqi, post-withdrawal, and 15/30 min post-withdrawal.

**Results:**

Following acupuncture at LI10, local SO_2_ at LI10 increased significantly (P<0.01), with transmission along the meridian to LI11 and LI8. No significant changes were observed at non-meridian non-acupoint sites.

**Conclusion:**

Acupuncture at Shousanli can specifically regulate blood oxygen metabolism at acupoint regions and demonstrates meridian-oriented transmission characteristics, providing visualized evidence for acupoint specificity.

## 1 Introduction

The meridian theory is at the core of traditional acupuncture, and. its essential features are the specificity of acupoints and the transmission through meridians. Whether these phenomena truly exist determines the reproducibility and scientific validity of acupuncture’s efficacy (Li et al., 2021; Liu et al., 2022; Gao et al., 2023; Kong et al., 2023). The transmission of acupuncture effects along meridian pathways after deqi achieved, which is a defining characteristic of meridian theory. Yet, visualizing and quantifying this process in living tissue has proven to be extremely difficult (Wang et al., 2021; Lin et al., 2022; Wang et al., 2024). Most current research relies on functional magnetic resonance imaging (fMRI), infrared thermography, and laser Doppler techniques, which have tentatively established that acupuncture can activate specific central functional networks and trigger local microcirculatory responses. However, these methods have the drawbacks of delayed temporal responses and the inability to integrate functional and structural observation, resulting gaps in the objective evidence chain connecting “deqi” with “arrival of qi at the disease site” (Si et al., 2021; Lu et al., 2025),thus fall short of precisely characterizing the dynamic progression of meridian transmission (Yoon et al., 2023; Valentini et al., 2024).

Photoacoustic imaging (PAI) is a novel hybrid modality that combines optical imaging’s high contrast with ultrasound’s deep tissue penetration and spatial resolution through a “light excitation-acoustic detection” mechanism (Park et al., 2022; Xu et al., 2022). By leveraging the distinct absorption peaks of oxygenated hemoglobin (HbO_2_) and deoxygenated hemoglobin (Hb) at specific wavelengths, PAI enables high-precision endogenous imaging using integrated photoacoustic algorithms. This permits non-invasive quantitative assessment of tissue oxygen saturation (SO_2_) without exogenous contrast agents, delivering both optical contrast and ultrasound penetration (Perekatova et al., 2019; Sarkar et al., 2023; Knieling et al., 2025). With real-time imaging capabilities at clinically relevant depths and spatial resolution around 50 μm, PAI shows considerable promise for integrated functional-structural observation (Kim et al., 2020; Park et al., 2023).

In recent years, multimodal PAI has found clinical applications in oncology and rheumatology, particularly evaluating tumor hypoxia and vascularization in joint inflammation (Lin and Wang, 2022; Huang et al., 2024).PAI can non-invasively visualize muscle oxygenation status (Karlas et al., 2020; Caranovic et al., 2025). However, its application in traditional Chinese medicine research remains limited, especially regarding validation of acupoint specificity and meridian transmission regularity, where substantial gaps persist (Wu et al., 2025).The present study employed PAI technology to track changes in blood oxygen metabolism within muscle tissues beneath the local acupoint and adjacent acupoints along the Hand-Yangming Large Intestine Meridian following acupuncture at Shousanli (LI10). Following WHO standards for acupoint localization (Lim, 2010), we adopted a randomized controlled design to compare differences between acupoints and non-meridian sites, and applied unsupervised cluster analysis to explore the spatiotemporal gradient characteristics of meridian transmission. Our goal was to establish SO_2_ spatiotemporal variation as a quantitative biomarker for meridian transmission, provide visualized functional imaging evidence for acupoint specificity, and promote the application of PAI in visualizing acupuncture research within traditional Chinese medicine.

## 2 Materials and methods

### 2.1 Study design

This study was designed as a single-center, randomized, single-blind (participant-blind), parallel-controlled clinical trial. It was divided into Acupoint group and Non-meridian group. Acupoint group: Right Shousanli (LI10), located on the dorsolateral aspect of the forearm, 6 cm below the elbow crease on the line connecting Yangxi and Quchi (LI11). Non-meridian group: Non-meridian non-acupoint site located 2 cm medial to LI10. The study adhered to the principles of the Declaration of Helsinki, and all participants provided written informed consent prior to enrollment.

Based on pilot data (n=8, mean ΔSO_2_=5.2%, SD = 3.8%), G*Power 3.1 calculation for independent t-test (two-tailed, α=0.05, power=0.80) yielded required n=18 per group. For repeated measures ANOVA (within-between interaction, f=0.25, α=0.05, power=0.80, 5 time points, correlation 0.5), required n=24 per group. We selected n=30 per group to reserve a 20% sample attrition buffer.

### 2.2 Participants

Inclusion criteria: (1) Healthy university students at Henan University of Chinese Medicine, aged 18–35 years, with body mass index (BMI) 18.5–26 kg/m²; (2) Right-handed individuals capable of tolerating acupuncture; (3) Non-smokers and non-heavy drinkers, with no acupuncture, massage, or other treatments within 2 weeks prior to the trial; (4) Voluntary participation with signed informed consent.

Exclusion criteria: (1) Pregnant or menstruating women; (2) Skin infection, ulceration, or dermatological conditions of the upper extremities; (3) Hematological disorders, bleeding tendencies, or coagulation dysfunction; (4) Severe cardiac, hepatic, or renal dysfunction; (5) History of neurological or psychiatric disorders; (6) History of upper extremity trauma or surgery within the past month; (7) History of needle fainting or fear of acupuncture.

**Figure 1:**
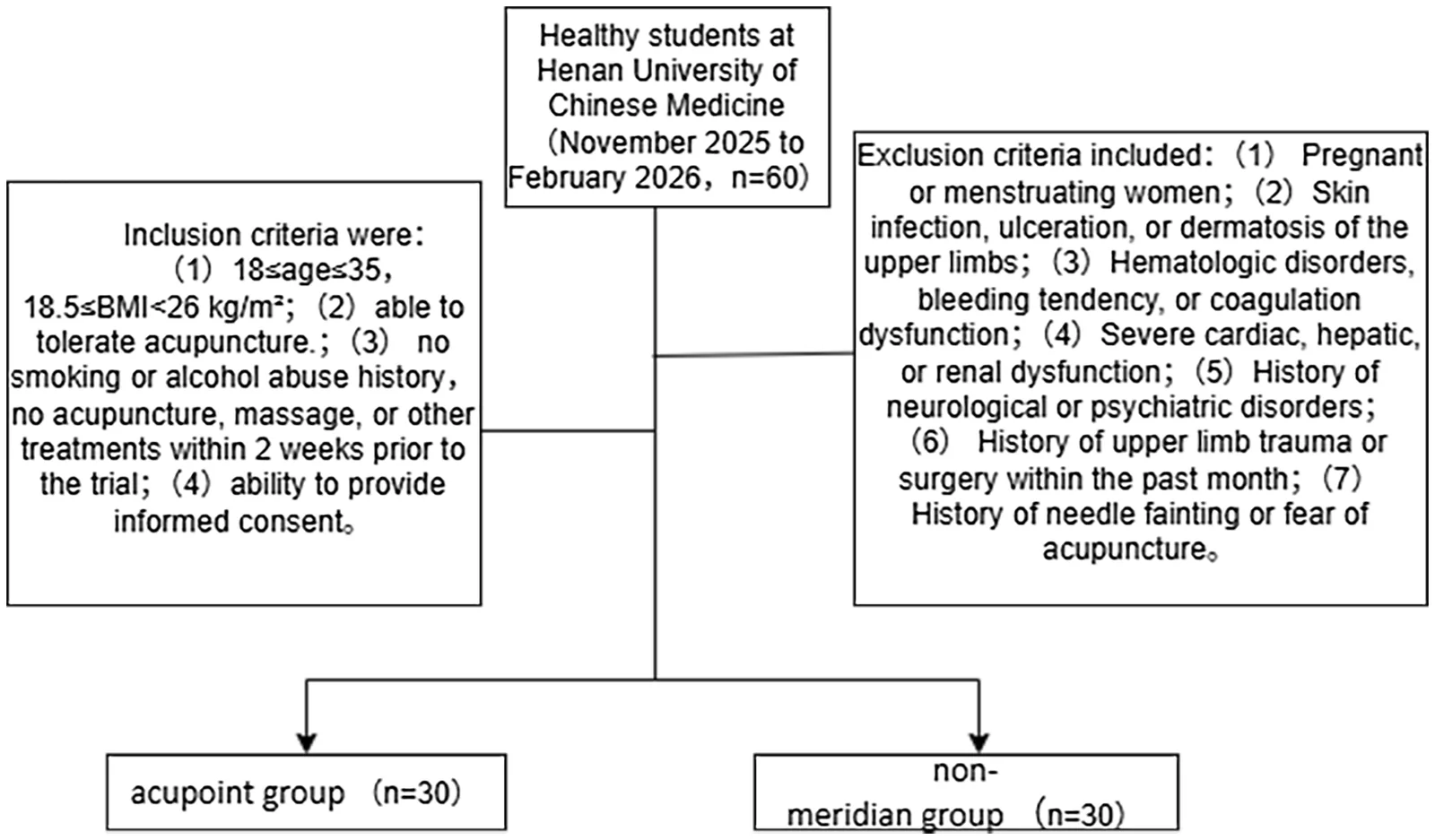
Flowchart of patient enrollment and study design. The enrollment flowchart delineates the patient selection trajectory: initial screening, exclusion criteria assessment, and final group assignment.

Withdrawal criteria: (1) Poor compliance with limb movement during detection resulting in data distortion; (2) Serious adverse reactions (e.g., needle fainting) during acupuncture; (3) Obvious deqi sensation in participants from the non-meridian group. The participant recruitment flowchart is presented in **Figure 1**.

### 2.3 Randomization and blinding

Randomization sequence was generated using SPSS 26.0 software by a statistician not involved in this study, with 60 participants allocated in a 1:1 ratio to the acupoint group and the non-meridian group. Randomization results were sealed in sequentially numbered, opaque envelopes and maintained by research personnel not involved in assessment or intervention. Upon enrollment, the study coordinator unsealed the envelope in sequence to inform the acupuncturist of group assignment. Given the nature of acupuncture manipulation, blinding of operators was not feasible; however, participants were blinded. To minimize communication bias, all participants were explicitly instructed not to discuss their acupuncture sensations or group assignment with other volunteers until completion of all observation time points.

### 2.4 Intervention

**Figure 2:**
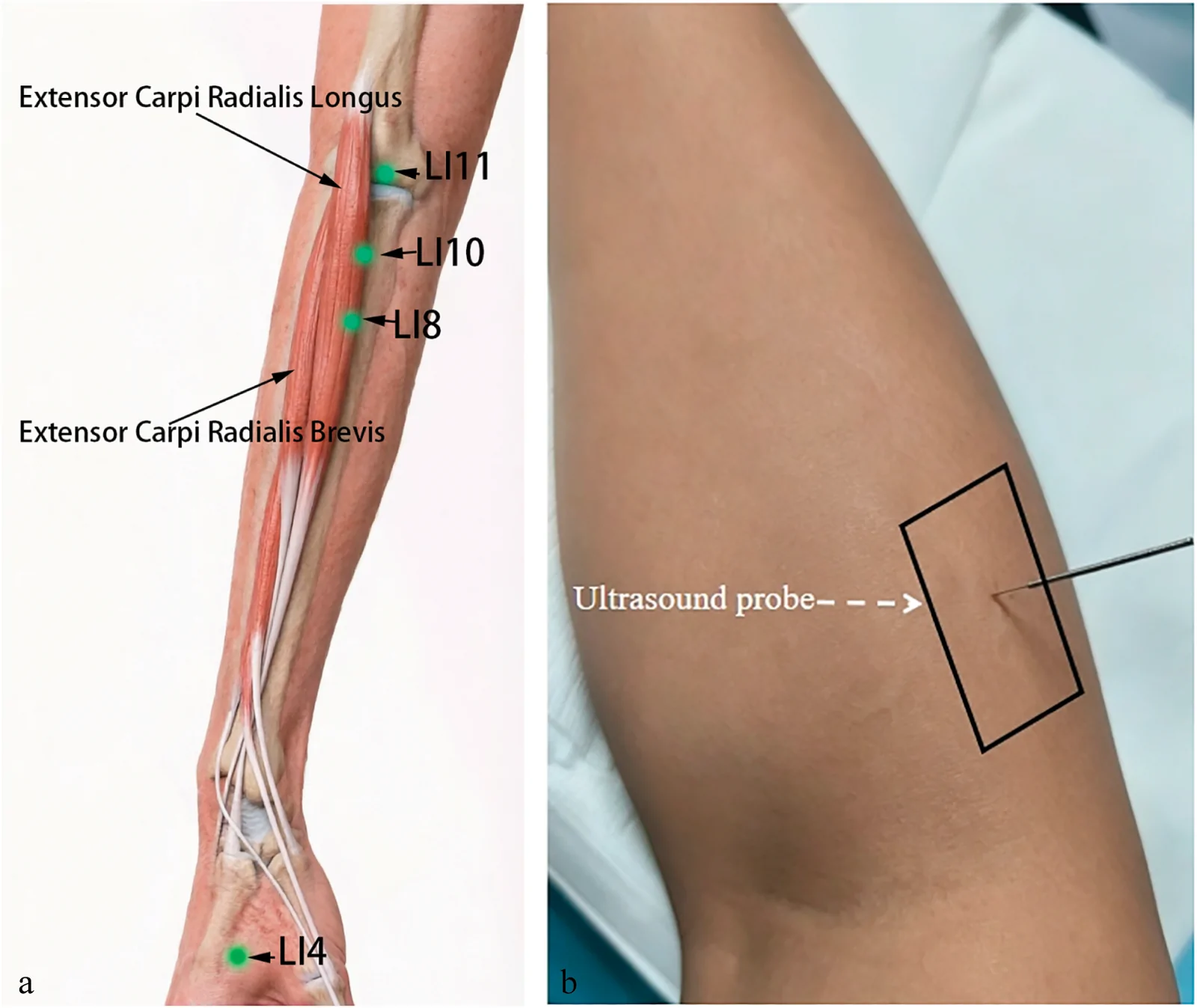
**(A)** Schematic diagram of acupoint localization. **(B)** shows the placement of linear PAI transducer, with the rectangular marker matching the probe’s coverage area and the acupuncture needle vertically penetrating LI10/non-meridian control point.

Acupoint localization: Following WHO standards (Lim, 2010), Shousanli (LI10) is located on the dorsolateral aspect of the forearm, on the line connecting Yangxi and Quchi (LI11), approximately 4cm below the elbow crease. Quchi (LI11) is located at the lateral end of the elbow crease, at the midpoint of the line connecting Chize and the lateral epicondyle of the humerus when the elbow is flexed. Xialian (LI8) is located 12 cm below the elbow crease on the line connecting Yangxi and LI11. Hegu (LI4) is located between the first and second metacarpal bones, at the midpoint of the second metacarpal bone on the radial side. Control points were located 2 cm medial to the meridian line at non-meridian non-acupoint sites (Zhou et al., 2021; Liu et al., 2023).The 2 cm medial site of LI10 was selected as non-meridian control for 3 core reasons: (1) avoiding meridian collateral pathways; (2) matched subcutaneous/muscle tissue composition with LI10, with no baseline SO_2_ difference; (3) consistent with mainstream acupuncture RCT non-acupoint offset standard (1.5–2 cm). The trivial ΔSO_2_ fluctuation (<0.6%) across all control sites verified this site can represent general non-meridian forearm tissue without meridian-specific oxygenation responses. The acupoint group received acupuncture at the right LI10, while the non-meridian group received acupuncture at a non-meridian non-acupoint site 2 cm medial to LI10. Photoacoustic imaging was subsequently performed to assess oxygen saturation (SO_2_) at multiple sites: in the acupoint group, measurements were taken at LI10, LI11, LI8, and LI4 along the Hand-Yangming Large Intestine Meridian; in the non-meridian group, corresponding non-meridian non-acupoint locations 2 cm medial to each respective acupoint were examined. The locations of the above-mentioned acupoints are shown in **Figure 2**. The rectangular marker (consistent with the probe footprint) was fully aligned with the needle insertion site to fix the PAI scanning plane, ensuring consistent ROI positioning across all subjects. The anatomical matching between LI10 and its medial control site as well as standard PAI probe placement are illustrated in **Figure 2**.

For acupuncture, disposable sterile filiform needles (0.30 mm × 40 mm, Huatuo Medical Instruments Co.,SuZhou, China) were used. Participants sat with the upper limb horizontal and the web of the hand facing upward. After routine skin disinfection, needles were inserted perpendicularly into LI10 to a depth of 15–20 mm. The reinforcing-reducing even method was applied through lifting-thrusting and twirling-rotating manipulation at 2 Hz frequency with 10 mm amplitude for 5 minutes, followed by 20 minutes needle retention. In the non-meridian group, needles were inserted into the non-meridian non-acupoint corresponding to LI10 to a depth of 4 mm without any manipulation or deqi induction, with 20 minutes retention. All procedures were performed by the same senior acupuncture physician.

It should be noted that this study did not include a placebo needle (sham) control group, based on the following considerations: (1) Streitberger placebo needles and similar devices are not widely available in China, and their simulation of skin penetration sensation remains debated (2) the core scientific question of this study was to validate ‘acupoint specificity’ rather than ‘acupuncture efficacy’ per se, and the non-meridian shallow needling group already partially controls for non-specific needling effects; (3) pilot data showed high baseline SO_2_ variability in placebo needle groups versus blank controls, potentially increasing required sample size. We acknowledge, however, that this design cannot fully distinguish ‘acupoint location effects’ from ‘needling manipulation intensity effects,’ which represents an important limitation discussed further below.

### 2.5 Photoacoustic imaging

Equipment: The Resona Y photoacoustic imaging ultrasound diagnostic system (Mindray Bio-Medical Electronics, ShenZhen, China) was employed, equipped with a handheld linear array transducer (5–10 MHz frequency, 128 elements) and a wavelength-tunable pulsed laser (750 nm and 830 nm, pulse width 4–7 ns, repetition frequency 10 Hz).

Scanning protocol: LI10, LI11, LI8, LI4, and control points were scanned at a depth of 35 mm. Regions of interest (ROI) were standardized at consistent depths, with each ROI measuring 5 mm × 20 mm. The ROI center was precisely positioned over each target acupoint based on anatomical landmarks, with the long axis aligned parallel to muscle fiber orientation to ensure the primary measurement region encompassed the core acupoint area. This ROI dimension was selected to accommodate standard ultrasound scanning protocols while ensuring adequate signal-to-noise ratio for photoacoustic spectral unmixing. Static frame scanning mode was employed, with continuous acquisition at each time point to obtain 3 frames.

**Figure 3:**
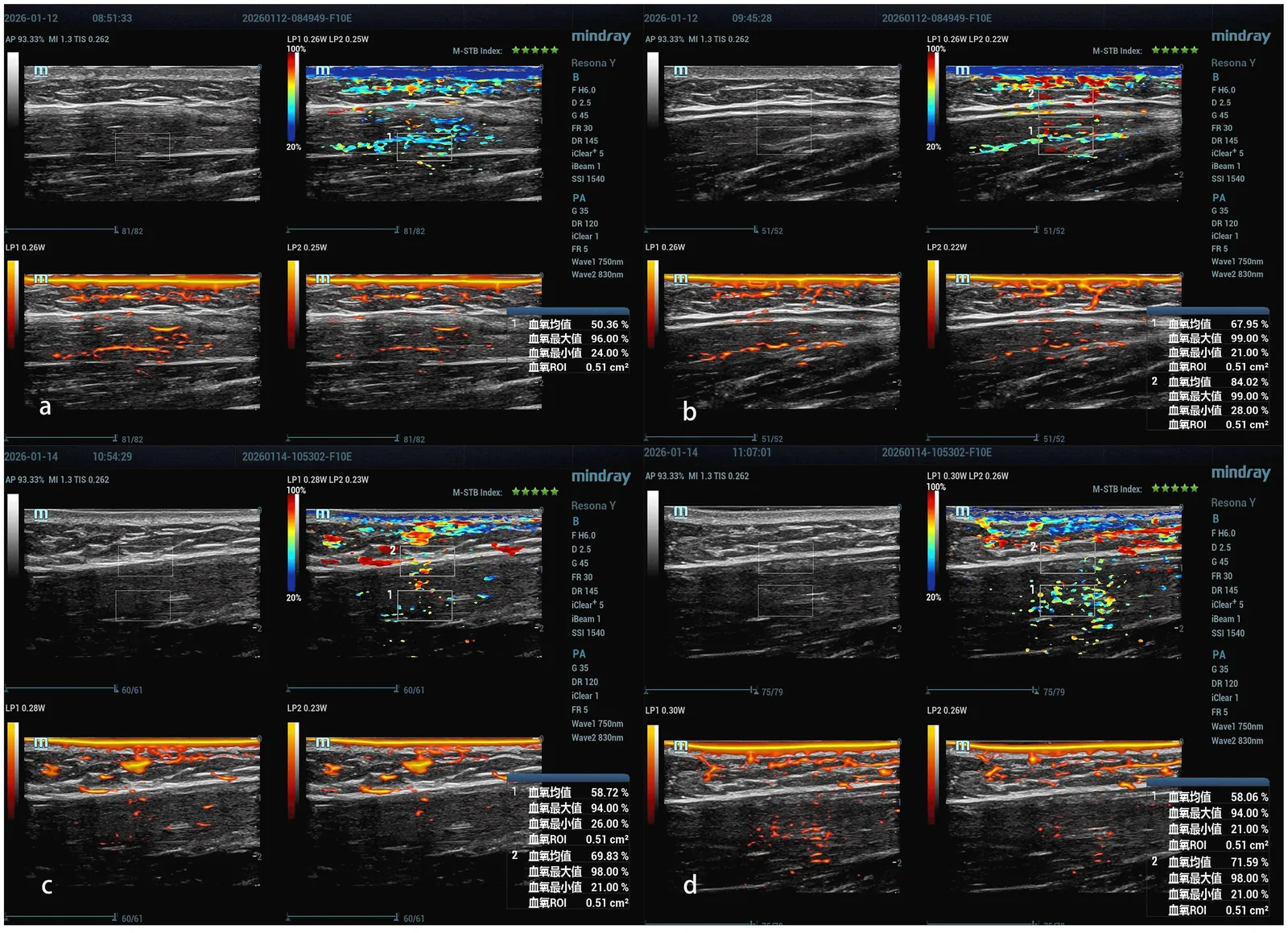
Upper left: Grayscale B-mode anatomical ultrasound for muscle layer identification (brachioradialis & extensor carpi radialis longus annotated). Upper right: Coregistered PAI pseudocolor SO_2_ functional heatmap (jet colormap: red = high oxygen saturation, blue = low saturation). Lower left: Raw grayscale photoacoustic signal intensity captured at 750 nm excitation wavelength for signal quality assessment. Lower right: Raw grayscale photoacoustic signal intensity captured at 830 nm excitation wavelength for signal quality assessment. **(a)** A vertical section of Shousanli (LI10) from a 21−year−old male volunteer. SO_2_ detected by PAI was 50.36%. **(b)** A vertical section of Shousanli (LI10) from a 21−year−old male volunteer. Post−acupuncture SO_2_ detected by PAI was 67.95%. **(c)** A vertical section of Shousanli (LI10) from a 20−year−old female volunteer.SO_2_ detected by PAI was 54.72%. **(d)** A vertical section of Shousanli (LI10) from a 20−year−old female volunteer. Post−acupuncture SO_2_ detected by PAI was 58.06%.Color scale represents SO_2_ percentage, with red/warm colors indicating higher oxygen saturation and blue/cool colors indicating lower oxygen saturation. Pixels with signal-to-noise ratio < 10 dB were excluded during ROI mean SO_2_ calculation to eliminate imaging noise interference.

Spectral unmixing: Dual-wavelength imaging at 750 nm (Hb absorption peak) and 830 nm (HbO_2_ absorption peak) was performed. SO_2_ was calculated using linear spectral unmixing algorithms as SO_2_ = HbO_2_/(HbO_2_ + Hb) × 100%, and total hemoglobin concentration (tHb = HbO_2_ + Hb) was determined through integrated algorithms based on photoacoustic signals. Assumptions for forearm muscle ROI: (1) Hb/HbO_2_ dominate photoacoustic signals; (2) water/lipid exhibit identical absorption at dual wavelengths, forming uniform background offset eliminated by ratio calculation; (3) epidermal melanin cannot penetrate deep muscle ROI and contributes no signal. The above dual-wavelength chromophore assumptions form the basis of all pixel-wise SO_2_ values visualized in representative multimodal PAI images (**Figure 3**).

**Figure 4:**
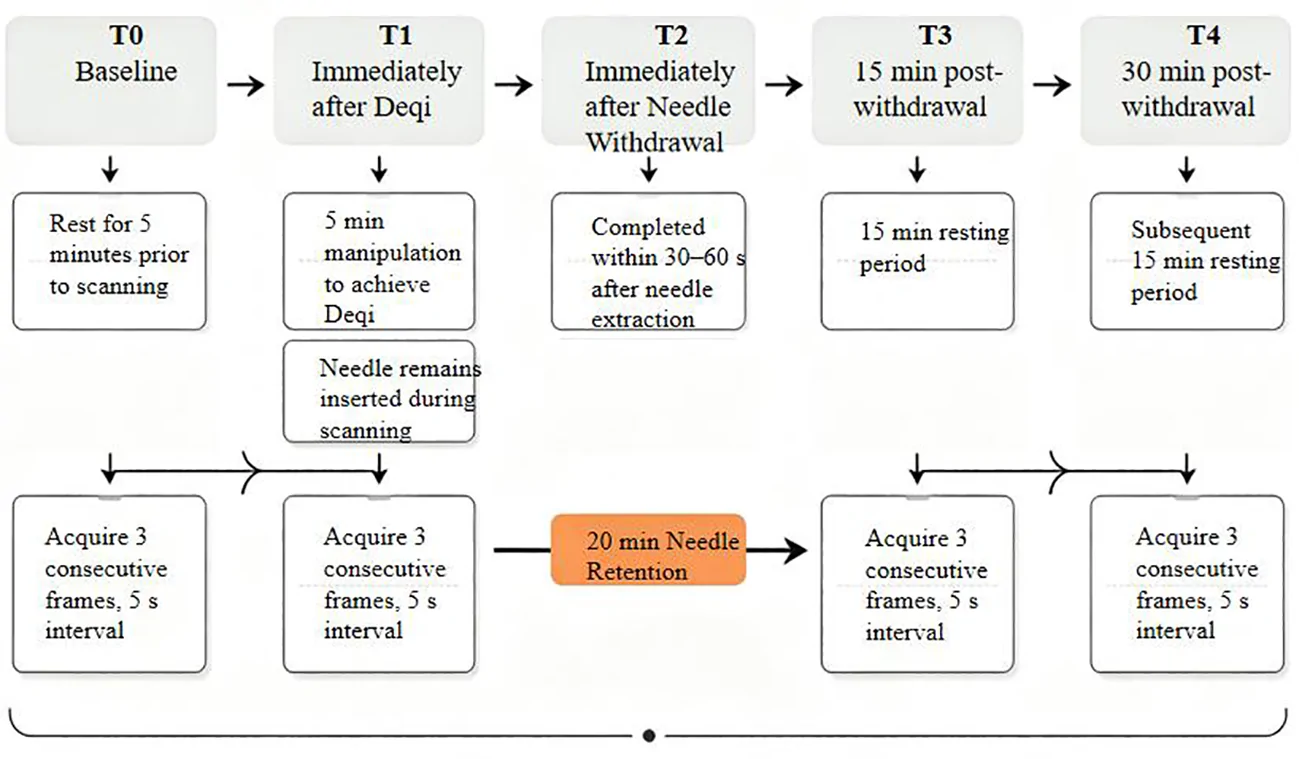
Timeline protocol for photoacoustic imaging acupuncture experiment.

Observation time points: T0 (baseline), T1 (immediately after deqi), T2 (immediately after needle withdrawal), T3 (15 min post-needle withdrawal), and T4 (30 min post-needle withdrawal). Participants rested for 5 min prior to each scanning session, with room temperature maintained at 25 ± 1 °C and humidity at 50%-60%.All scanning timings and frame acquisition rules are standardized in **Figure 4** Timeline Protocol for repeatable measurement.

### 2.6 Image assessment

Multimodal photoacoustic/ultrasound (PA/US) imaging was performed by two senior sonographers at each point. Standard B-mode ultrasound was first employed to observe muscle morphology beneath acupoints, followed by PAI assessment of muscle SO_2_ in the longitudinal plane. Three measurements were obtained at each site, with mean SO_2_ values calculated. Inter-observer agreement was assessed using intraclass correlation coefficient (ICC), with excellent consistency achieved (ICC > 0.90).Scattered colored pixels inside the ROI represent pixel-wise SO_2_ solved via linear spectral unmixing; only the averaged value of pixels with SNR ≥ 10 dB within the 5 mm × 20 mm ROI was adopted for statistical analysis, while low-SNR signals were discarded to eliminate imaging noise. Representative images are presented in **Figure 3**.

### 2.7 Outcome measures

Primary outcomes: SO_2_ and ΔSO_2_ (ΔSO_2_ = SO_2_ at each time point - baseline SO_2_) at each point.

### 2.8 Statistical analysis

All statistical analyses were conducted with SPSS 26.0 and Python 3.10 (pingouin package).

Shapiro-Wilk test was used to assess normality of SO_2_ data. Mauchly’s test was applied to evaluate sphericity assumption; Greenhouse-Geisser correction was adopted when sphericity was violated. Accordingly, corrected degrees of freedom will be reported for all repeated−measures effects.

Two-way mixed-design repeated-measures ANOVA was separately performed for each acupoint, with Group (acupoint vs. non-meridian control) as the between-subject factor and Time (5 time points) as the within-subject factor. Hierarchical Bonferroni-corrected *post-hoc* pairwise comparisons were conducted following significant main or interaction effects to examine:

Intra-group longitudinal comparisons: SO_2_ at each time point versus baseline within each group;

Inter-group horizontal comparisons: ΔSO_2_ differences between the two groups at each identical time point.

Friedman test and Mann-Whitney U test were employed as non-parametric sensitivity analyses for datasets deviating from normal distribution. Intraclass correlation coefficient (ICC) was calculated to evaluate inter-observer measurement consistency. All tests were two-sided, and adjusted P < 0.05 was considered statistically significant.

## 3 Results

### 3.1 Baseline characteristics

A total of 60 volunteers were recruited, with 30 participants in each group. The groups were balanced regarding sex distribution (males: 51.7% vs. 48.3%, χ² = 0.07, P = 0.80), age (22 [20, 24] years vs. 22 [20.75, 25] years, t = -0.56, P = 0.58), and BMI (21 [20, 22.25] vs. 21 [20, 23.25], t = -0.30, P = 0.76), with no statistically significant differences (P > 0.05), indicating good baseline comparability (**Table 1**).

**Table 1:** Baseline characteristics comparison between two subject groups.

Variables	Non-meridian group (n=30)	Acupoint group (n=30)	t	p
Age	22(20, 24)	22(20.75, 25)	-0.56	0.58
BMI	21(20, 22.25)	21(20, 23.25)	-0.30	0.76
Gender(males)	(51.7%)	(48.3%)		

### 3.2 Local effects of acupuncture at Shousanli on blood oxygen (acupoint specificity)

Mixed-design repeated measures ANOVA revealed significant main effects of Time (F(0.304,17.632) = 18.47, p < 0.001, η²p = 0.242) and Group (F(1,58) = 45.23, p < 0.001, η²p = 0.438), and a significant Time × Group interaction (F0.304,17.632) = 12.36, p < 0.001, η²p = 0.176).Shapiro-Wilk normality test (**Supplementary Table 1**) indicated partial datasets deviated from normal distribution; non-parametric Friedman and Mann-Whitney U tests (**Supplementary Table 6**, **S7**) were performed as sensitivity analyses to verify the robustness of parametric results.

Simple effect analysis with Bonferroni correction (α = 0.0025) demonstrated that in the acupoint group, SO_2_ at LI10 increased significantly from baseline at all post-intervention time points: T1 (ΔSO_2_ = 5.60 ± 4.01%, p < 0.001), T2 (8.00 ± 3.91%, p < 0.001), T3 (8.23 ± 5.12%, p < 0.001), and T4 (9.33 ± 4.79%, p < 0.001). The effect size was large (Cohen’s d = 1.39 at T1, 95% CI: 1.15–2.29). In stark contrast, the non-meridian group exhibited negligible fluctuations in ΔSO_2_ at the corresponding control site, with absolute changes less than 0.6% at all time points; none of these minor deviations reached statistical significance versus baseline (all P > 0.05).

Between-group comparisons further demonstrated that ΔSO_2_ magnitudes at LI10 were markedly higher in the acupoint group than in the non-meridian group at every observation time point (all Bonferroni-adjusted P < 0.001, marked with # in **Table 2**). Inter-site pairwise comparisons (LI10 vs LI11/LI8/LI4) were additionally conducted to assess the spatial gradient of oxygenation responses. At all time points, ΔSO_2_ of LI10 was significantly greater than that of LI8. No statistically significant inter-site differences were detected between LI10 and LI11 at any time point, while LI4 yielded comparable or even larger ΔSO_2_ values than LI10 across all time windows. Collectively, these results validate the specific regulatory effect of LI10 acupuncture on local tissue blood oxygen metabolism (**Table 2**).

**Table 2:** Comparison of ΔSO_2_ changes at different time points between groups (mean ± SD).

Group	Non-meridian group ΔSO_2_ (95%CI)	Acupoint group ΔSO_2_ (95%CI)
SO_2_		
Shousanli (LI10) Deqi	0.53 ± 1.80(−0.14,1.20)	5.60 ± 4.01(4.10,7.10) ^*#^
Shousanli (LI10) Needle withdrawal	0.27 ± 1.60(−0.33,0.87)	8.00 ± 3.91(6.54,9.46)^* #^
Shousanli (LI10) 15 min	0.37 ± 1.38(−0.15,0.89)	8.23 ± 5.12(6.32,10.14)^* #^
Shousanli (LI10)30min	-0.07 ± 1.44(−0.61,0.47)	9.33 ± 4.79(7.54,11.12) ^*#^
Quchi (LI11) Deqi	0.25 ± 2.47(−0.67,1.17)	5.39 ± 5.79(3.23,7.55) ^*#^
Quchi (LI11) Needle withdrawal	0.01 ± 2.60(−0.96,0.98)	6.62 ± 4.06(5.10,8.14) ^*#^
Quchi (LI11) 15min	0.13 ± 1.41(−0.39,0.65)	7.23 ± 5.02(5.36,9.10) ^*#^
Quchi (LI11) 30min	-0.03 ± 1.52(−0.59,0.53)	7.37 ± 4.67(5.63,9.11) ^*#^
Xialian (LI8) Deqi	0.48 ± 1.74(−0.17,1.13)	3.95 ± 5.42(1.93,5.97) ^*#^
Xialian (LI8) Needle withdrawal	-0.15 ± 0.69(−0.41,0.11)	5.72 ± 6.56(3.28,8.16) ^*#^
Xialian (LI8) 15min	-0.19 ± 1.94(−0.91,0.53)	5.18 ± 6.95(2.59,7.77) ^*#^
Xialian (LI8) 30min	-0.29 ± 0.79(−0.59,0.01)	7.68 ± 5.99(5.45,9.91) ^*#^
Hegu (LI4) Deqi	-0.87 ± 0.70(−1.13,−0.61)	6.23 ± 3.76(4.83,7.64) ^*#^
Hegu(LI4)Needle withdrawal	-0.23 ± 0.95(−0.58,0.12)	10.23 ± 4.13(8.69,11.78) ^*#^
Hegu (LI4)15min	-0.40 ± 1.03(−0.78,0.02)	11.03 ± 4.87(9.22,12.85) ^*#^
Hegu (LI4)30min	-0.80 ± 0.70(−1.06,−0.54)	9.83 ± 5.65(7.73,11.94) ^*#^

ΔSO_2_ = SO_2_ value at each time point minus baseline SO_2_. All data are presented as mean ± standard deviation (95% confidence interval in parentheses). Symbol annotations: *Significant difference versus baseline within the acupoint group after hierarchical Bonferroni correction (adjusted P < 0.0025); ^#^Significant between-group difference at the identical time point, Bonferroni-adjusted P < 0.0025; the mixed-design repeated-measures ANOVA performed separately for each acupoint, Mauchly’s test of sphericity revealed that all acupoints violated the sphericity assumption (**Supplementary Table 2**), thus Greenhouse-Geisser correction was uniformly applied to all acupoints.

When interpreting these between-group differences, it is important to note that because the non-meridian group did not receive lifting-thrusting/twirling-rotating manipulation, the observed effects likely comprise two contributions: (a) specific physiological responses of the LI10 acupoint itself, and (b) non-specific neuro-humoral reactions elicited by deqi-inducing manipulation. Therefore, the conclusion of ‘acupoint specificity’ should be understood as a combined effect of ‘specific acupoint + deqi manipulation’ rather than purely anatomical location effects.

### 3.3 Meridian transmission analysis

**Figure 5:**
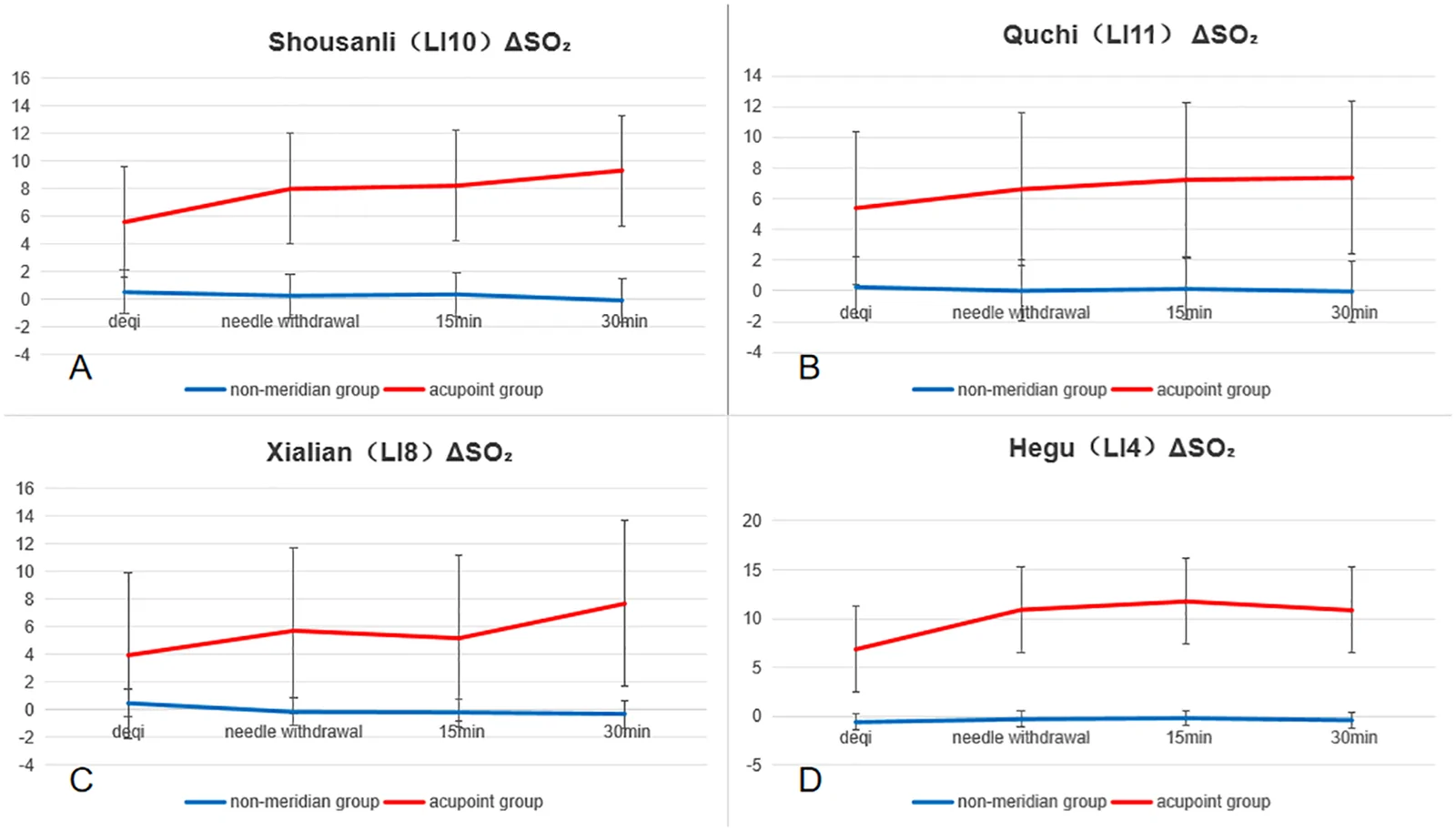
Time-course curves of SO_2_ changes at acupuncture points in both groups. Vertical capped bars represent 95% confidence intervals. Red solid circles = acupoint group; hollow blue squares = non-meridian control group. **(A)** SO_2_ change trend at Shousanli (LI10); **(B)** SO_2_ change trend at Quchi (LI11); **(C)** SO_2_ change trend at Xialian (LI8); **(D)** SO_2_ change trend at Hegu (LI4).

Mixed-design repeated-measures ANOVA (global model, **Supplementary Table 3**) detected significant Time × Group interaction effects for LI11, LI8 and LI4: LI11 (F(0.304,17.632) = 18.334, P < 0.001, η²p = 0.240), LI8 (F(0.312,18.096) = 12.439, P < 0.001, η²p = 0.177), and LI4 (F(0.264,15.312) = 57.215, P < 0.001, η²p = 0.497).Bonferroni-corrected simple effect analyses confirmed consistent, significant SO_2_ elevation from baseline at all time points for LI11, LI8 and LI4 in the acupoint group (all adjusted P < 0.0025), while no meaningful SO_2_ fluctuations were observed in the corresponding non-meridian control sites across the entire observation window. The non-overlapping 95% confidence interval error bars in **Figure 5** visually confirm the significant intergroup differences consistent with mixed-design repeated-measures ANOVA results shown in **Table 2**.

Spatial distribution of ΔSO_2_ across the Hand-Yangming Large Intestine Meridian revealed a proximal-to-distal declining gradient at T1–T3 (LI10 > LI11 > LI8), consistent with distance-dependent attenuation of acupuncture-induced meridian conduction signals. At T4 (30 min post-withdrawal), ΔSO_2_ of LI8 (7.68 ± 5.99%) slightly exceeded that of LI11 (7.37 ± 4.67%); this minor crossover represents transient individual variation rather than a full reversal of the overall decreasing spatial gradient. This delayed distal elevation may be explained by prolonged transmission latency of meridian qi toward distal segments, or inter-subject variability in microvascular response kinetics at LI8. Critically, this transient fluctuation does not override the dominant proximal-stronger spatial pattern identified at T1–T3.Notably, the Yuan-source acupoint LI4 exhibited larger ΔSO_2_ magnitudes than LI11 and LI8 at all four time points (T1: 6.23% vs. 5.39% and 3.95%; T2: 10.23% vs. 6.62% and 5.72%; T3: 11.03% vs. 7.23% and 5.18%; T4: 9.83% vs. 7.37% and 7.68%). This consistent amplified response at LI4 aligns with classical meridian theory stating that Yuan-source acupoints serve as primary convergence hubs for channel qi, and exhibit heightened sensitivity to upstream acupuncture stimulation. Descriptively, ΔSO_2_ values at LI10 were numerically higher than those at LI8 at all time points (T1: 5.60% vs. 3.95%; T2: 8.00% vs. 5.72%; T3: 8.23% vs. 5.18%; T4: 9.33% vs. 7.68%), consistent with a proximal-to-distal declining oxygenation gradient along the Hand-Yangming Large Intestine Meridian.

The temporal dynamic curves of ΔSO_2_ for all four acupoints are visualized in **Figure 5**.

## 4 Discussion

### 4.1 Principal findings

This study marks the first application of PAI technology to visualize acupoint specificity and meridian transmission following acupuncture at Shousanli.

We found that acupuncture specifically increased SO_2_ at the LI10 region and transmitted along the Hand-Yangming Large Intestine Meridian toward LI11, LI8, and LI4, forming a proximal-to-distal decreasing gradient (LI10 > LI11 > LI8) with sustained temporal elevation. These findings provide objective blood oxygen metabolism evidence for “deqi” and “meridian sensation transmission,” supporting the objective existence of meridians as functional conduction pathways.

The robustness of these conclusions is strengthened by: (a) strict Bonferroni correction for 20 comparisons (α =0.0025), with all primary findings remaining significant; (b)Greenhouse-Geisser correction was uniformly adopted for all acupoints; and (c) non-parametric sensitivity analyses confirming the stability of results (**Supplementary Materials**).

### 4.2 Comparison with previous studies

Earlier research predominantly used laser Doppler to monitor skin microcirculation or fMRI to observe central activation, but could not simultaneously achieve precise localization of deep tissue function and structure (Zhuang et al., 2022; Chu et al., 2025). The PAI technology in this study combines 50 μm-level spatial resolution with real-time dynamic monitoring, clearly displaying oxygenation changes in muscle tissues up to 35 mm depth following acupuncture. Compared with ultrasound contrast technology, PAI more directly reflects muscle metabolic levels; compared with near-infrared spectroscopy, PAI offers superior anatomical localization precision (Karagozoglu et al., 2025; Wu et al., 2025). A recent shear wave elastography study found that acupuncture at Hegu could alter local tissue stiffness (Dai et al., 2020); our study complements this evidence by demonstrating that acupuncture at Hegu not only alters local tissue stiffness but also enhances regional blood oxygen metabolism, thereby providing functional evidence of improved microcirculatory perfusion from the blood oxygen perspective.

The non-meridian shallow needling control used here is methodologically superior to no-treatment controls but inferior to placebo needle controls. Langevin et al. (2001) systematic review noted that non-meridian shallow needling may still activate cutaneous mechanoreceptors, producing mild peripheral effects; Streitberger and Kleinhenz (1998)found that placebo needles better control for psychological expectancy but may blunt or underestimate true acupuncture efficacy by eliminating tactile afferent input. The minimal SO_2_ changes in our non-meridian group (|ΔSO_2_|<0.6%) suggest limited physiological interference from shallow needling, supporting that the main effects arise from acupoint-manipulation interactions. Future studies should adopt three-arm designs (verum acupuncture, placebo needle, non-meridian) to independently parse these effects.

### 4.3 Mechanistic considerations

The attainment of deqi through acupuncture may regulate blood oxygen metabolism through several mechanisms: (1) Activation of Group II/III afferent nerve fibers at acupoint regions releases vasoactive substances such as calcitonin gene-related peptide (CGRP) and nitric oxide (NO) through axon reflexes, causing local microvascular dilation (Fan et al., 2023); (2) Triggering sympathetic-parasympathetic balance regulation increases capillary opening density (Valentini et al., 2024). The blood oxygen elevation along meridian directions observed in this study supports the neuro-humoral coupling hypothesis. The 5–10% absolute SO_2_ increase observed in our study corresponds to an estimated 15–25% increase in tissue perfusion, consistent with laser Doppler findings, and aligns with the 2–5 second latency of axon reflexes at T1 (immediate post-deqi). The sustained elevation through T4 suggests secondary metabolic hyperemia via adenosine and NO-mediated vasodilation. At the molecular mechanism level, we hypothesize that CGRP release activates endothelial eNOS-NO pathways causing microvascular dilation; concurrently, acupuncture-induced β-endorphin release may modulate sympathetic tone via central descending inhibitory pathways, reducing vascular smooth muscle contraction. The SO_2_ elevation detected by PAI likely reflects summation of these peripheral and central mechanisms. However, this study cannot distinguish their relative contributions: if effects primarily arise from local axon reflexes, they should disappear in denervated tissue; if mainly from central modulation, mirror effects should occur in the contralateral limb. These predictions can be tested in future studies combining microdialysis (for local CGRP/NO concentrations) or crossover experimental designs.

From the meridian theory perspective, the Hand-Yangming Large Intestine Meridian “originates from the radial side of the index finger and ascends along the anterolateral aspect of the upper limb.” In this study, LI4 (Hegu), as the Yuan-source point of the Large Intestine Meridian, showed blood oxygen response magnitudes exceeding those of LI11 and LI8. This phenomenon can be understood in three ways: First, Hegu represents the convergence of qi and blood of the Large Intestine Meridian, making it more sensitive to changes in channel qi; second, acupuncture at LI10 may activate specific responses at the distal Yuan-source point through meridian-organ connections; third, this result corresponds with the clinical therapeutic principle of “treating disorders of the face and mouth with Hegu,” suggesting the pivotal role of Yuan-source points in channel qi transmission. Additionally, the decreasing gradient pattern of blood oxygen from LI10→LI11→LI8 observed here does not fully align with the “circular and endless” flow direction of channel qi, which may relate to the stronger local effects of acupuncture compared to distal transmission, or may reflect specific patterns of qi and blood flow in the Hand-Yangming Meridian from elbow to wrist—an area warranting further investigation. The transient distal surpassing at LI8 at T4 may indicate dynamic redistribution of meridian qi following initial local accumulation.

### 4.4 Clinical implications

This study establishes a novel functional imaging approach for acupoint evaluation, providing evidence for precision acupuncture in clinical practice. Future applications may extend to pathological conditions such as upper extremity pain or post-stroke hemiplegia to explore abnormal metabolic characteristics of diseased meridians, and may combine with focused ultrasound for non-invasive acupoint regulation and efficacy assessment.

Future directions for PAI technology in acupuncture research include: (1) Meridian-organ correlation studies: monitoring effects of acupuncture at specific acupoints on blood oxygen metabolism in target organs to validate “meridian-organ” connections; (2) Disease state assessment: comparing acupoint response characteristics between healthy individuals and patients (e.g., post-stroke hemiplegia, diabetic peripheral neuropathy) to establish diagnostic and predictive models; (3) Acupuncture parameter optimization: systematically comparing effects of different manipulation techniques, depths, and retention times on SO_2_ dynamic curves to develop individualized acupuncture protocols; (4) Multimodal integration: combining PAI with fMRI, and other technologies to construct comprehensive research systems from peripheral acupoints to central responses.

## 5 Conclusion

Acupuncture at Shousanli specifically enhances local tissue oxygen saturation and demonstrates spatiotemporally decreasing transmission along the Hand-Yangming Large Intestine Meridian. Photoacoustic imaging provides visualized and quantifiable functional imaging evidence for acupoint specificity and meridian transmission, representing a powerful tool for the modernization of meridian research.

## 6 Limitations

This study only recruited healthy young adults aged 18–35 years, limiting the generalizability of our results to elderly subjects and patients with endothelial dysfunction. Future multi-center trials will enroll participants of varying ages and clinical statuses for external validation. Non-meridian shallow needling was used instead of standard placebo needles, which fails to eliminate psychological expectancy bias induced by deqi and may overestimate acupoint-specific effects. The 30-minute observation window only captured short-term microcirculatory changes, lacking long-term physiological data of acupuncture. Extended follow-up to 2 h and 24 h will be adopted in subsequent studies to describe sustained meridian oxygenation responses. PAI only reaches a maximum depth of 35 mm and cannot detect deep acupoints. Depth-related signal attenuation may interfere with the observed meridian oxygen gradient; multi-frequency PAI or combined imaging modalities will be applied to separate technical artifacts from biological responses. Bonferroni correction used for multiple comparisons is overly conservative for repeated-measures data and raises Type II error risk. FDR correction will be implemented in follow-up analyses to optimize statistical reliability.

## Data Availability

The raw data supporting the conclusions of this article will be made available by the authors, without undue reservation.
