# Supplementary material for: Photoacoustic imaging of acupoint specificity and meridian transmission at Shousanli (LI10): a blood oxygen metabolism study
Source: Front Physiol. 2026 Aug 21;17:1911653. doi: 10.3389/fphys.2026.1911653 (PMC13541443; doi:10.3389/fphys.2026.1911653)
Supplement: Supplementary file 1 [file Table1.docx]

Acu = Acupoint group. Non-mer = Non-meridian group. *: P < 0.0025 after Bonferroni.

W = Shapiro-Wilk statistic. Normality assumed when P > 0.05.

| Acupoint | Time | Acu W | Acu P | Normal | Non-mer W | Non-mer P | Normal |
| --- | --- | --- | --- | --- | --- | --- | --- |
| LI10 | Baseline | 0.9428 | 0.1080 | Yes | 0.9533 | 0.2068 | Yes |
| LI10 | Deqi | 0.9708 | 0.5621 | Yes | 0.9387 | 0.0838 | Yes |
| LI10 | Remove | 0.9549 | 0.2284 | Yes | 0.9578 | 0.2712 | Yes |
| LI10 | 15min | 0.9737 | 0.6452 | Yes | 0.9550 | 0.2293 | Yes |
| LI10 | 30min | 0.9445 | 0.1201 | Yes | 0.9453 | 0.1263 | Yes |
| LI11 | Baseline | 0.9039 | 0.0105 | No | 0.9481 | 0.1508 | Yes |
| LI11 | Deqi | 0.8958 | 0.0066 | No | 0.9595 | 0.3004 | Yes |
| LI11 | Remove | 0.9198 | 0.0265 | No | 0.9382 | 0.0814 | Yes |
| LI11 | 15min | 0.9589 | 0.2907 | Yes | 0.9200 | 0.0268 | No |
| LI11 | 30min | 0.9637 | 0.3846 | Yes | 0.9665 | 0.4493 | Yes |
| LI8 | Baseline | 0.9432 | 0.1111 | Yes | 0.9624 | 0.3568 | Yes |
| LI8 | Deqi | 0.9656 | 0.4272 | Yes | 0.9779 | 0.7676 | Yes |
| LI8 | Remove | 0.9678 | 0.4807 | Yes | 0.9708 | 0.5612 | Yes |
| LI8 | 15min | 0.9064 | 0.0121 | No | 0.9690 | 0.5121 | Yes |
| LI8 | 30min | 0.9696 | 0.5294 | Yes | 0.9695 | 0.5254 | Yes |
| LI4 | Baseline | 0.9688 | 0.5075 | Yes | 0.9070 | 0.0125 | No |
| LI4 | Deqi | 0.9648 | 0.4089 | Yes | 0.8991 | 0.0080 | No |
| LI4 | Remove | 0.9632 | 0.3732 | Yes | 0.8743 | 0.0021 | No |
| LI4 | 15min | 0.9552 | 0.2325 | Yes | 0.9173 | 0.0229 | No |
| LI4 | 30min | 0.9600 | 0.3099 | Yes | 0.9217 | 0.0297 | No |

**Supplementary Table S1. Shapiro-Wilk Normality Test**
