# Supplementary material for: Photoacoustic imaging of acupoint specificity and meridian transmission at Shousanli (LI10): a blood oxygen metabolism study
Source: Front Physiol. 2026 Aug 21;17:1911653. doi: 10.3389/fphys.2026.1911653 (PMC13541443; doi:10.3389/fphys.2026.1911653)
Supplement: Supplementary file 2 [file Table2.docx]

*GG = Greenhouse-Geisser; HF = Huynh-Feldt.*

| Acupoint | Mauchly's W | χ² | df | P | GG ε | HF ε | Correction |
| --- | --- | --- | --- | --- | --- | --- | --- |
| LI10 | 0.4234 | 49.352 | 9 | <0.001 | 0.076 | 0.087 | GG correction |
| LI11 | 0.5039 | 39.352 | 9 | <0.001 | 0.076 | 0.087 | GG  correction |
| LI8 | 0.4582 | 44.814 | 9 | <0.001 | 0.078 | 0.089 | GG correction |
| LI4 | 0.1240 | 119.861 | 9 | <0.001 | 0.066 | 0.074 | GG correction |

**Supplementary Table S2. Mauchly's Test of Sphericity**
