# Supplementary material for: Photoacoustic imaging of acupoint specificity and meridian transmission at Shousanli (LI10): a blood oxygen metabolism study
Source: Front Physiol. 2026 Aug 21;17:1911653. doi: 10.3389/fphys.2026.1911653 (PMC13541443; doi:10.3389/fphys.2026.1911653)
Supplement: Supplementary file 3 [file Table3.docx]

*T×G = Time × Group interaction. η²p = partial eta squared.*

| Acupoint | Time F | Time df | Time P | Time η²p | Group F | Group df | Group P | Group η²p | T×G F | T×G df | T×G P | T×G η²p |
| --- | --- | --- | --- | --- | --- | --- | --- | --- | --- | --- | --- | --- |
| LI10 | 30.541 | 0.304, 17.634 | <0.001 | 0.3449 | 0.131 | 1, 58 | 0.7192 | 0.0022 | 29.141 | 0.304, 17.634 | <0.001 | 0.3344 |
| LI11 | 18.334 | 0.304, 17.634 | <0.001 | 0.2402 | 1.050 | 1, 58 | 0.3097 | 0.0178 | 17.944 | 0.304, 17.634 | <0.001 | 0.2363 |
| LI8 | 12.439 | 0.312, 19.096 | <0.001 | 0.1766 | 0.062 | 1, 58 | 0.8050 | 0.0011 | 14.908 | 0.312, 19.096 | <0.001 | 0.2045 |
| LI4 | 57.215 | 0.264, 15.312 | <0.001 | 0.4966 | 0.900 | 1, 58 | 0.3466 | 0.0153 | 65.713 | 0.264, 15.312 | <0.001 | 0.5312 |

All reported degrees of freedom for within‑subject factors have been adjusted using the Greenhouse–Geisser epsilon.

**Supplementary Table S3. Repeated Measures ANOVA Results**
