# Supplementary material for: Photoacoustic imaging of acupoint specificity and meridian transmission at Shousanli (LI10): a blood oxygen metabolism study
Source: Front Physiol. 2026 Aug 21;17:1911653. doi: 10.3389/fphys.2026.1911653 (PMC13541443; doi:10.3389/fphys.2026.1911653)
Supplement: Supplementary file 4 [file Table4.docx]

*BL = Baseline. Bonf.P = Bonferroni-adjusted P. *: P < 0.0025.*

| Acupoint | Comparison | MeanΔ | SD | CI Low | CI High | t | df | Unadj.P | Cohen's d | Bonf.P | Sig. |
| --- | --- | --- | --- | --- | --- | --- | --- | --- | --- | --- | --- |
| LI10 | Deqi vs BL | 5.60 | 4.01 | 4.10 | 7.10 | 7.642 | 29 | <0.001 | 1.395 | <0.001 | * |
| LI10 | Remove vs BL | 8.00 | 3.91 | 6.54 | 9.46 | 11.198 | 29 | <0.001 | 2.045 | <0.001 | * |
| LI10 | 15min vs BL | 8.23 | 5.12 | 6.32 | 10.14 | 8.813 | 29 | <0.001 | 1.609 | <0.001 | * |
| LI10 | 30min vs BL | 9.33 | 4.79 | 7.54 | 11.12 | 10.662 | 29 | <0.001 | 1.947 | <0.001 | * |
| LI11 | Deqi vs BL | 5.39 | 5.79 | 3.22 | 7.55 | 5.096 | 29 | <0.001 | 0.930 | <0.001 | * |
| LI11 | Remove vs BL | 6.62 | 4.06 | 5.10 | 8.13 | 8.928 | 29 | <0.001 | 1.630 | <0.001 | * |
| LI11 | 15min vs BL | 7.23 | 5.02 | 5.36 | 9.11 | 7.889 | 29 | <0.001 | 1.440 | <0.001 | * |
| LI11 | 30min vs BL | 7.37 | 4.67 | 5.62 | 9.11 | 8.637 | 29 | <0.001 | 1.577 | <0.001 | * |
| LI8 | Deqi vs BL | 3.95 | 5.42 | 1.93 | 5.97 | 3.991 | 29 | 0.0004 | 0.729 | 0.0016 | * |
| LI8 | Remove vs BL | 5.72 | 6.56 | 3.27 | 8.17 | 4.771 | 29 | <0.001 | 0.871 | <0.001 | * |
| LI8 | 15min vs BL | 5.18 | 6.95 | 2.59 | 7.78 | 4.084 | 29 | 0.0003 | 0.746 | 0.0012 | * |
| LI8 | 30min vs BL | 7.68 | 5.99 | 5.45 | 9.92 | 7.027 | 29 | <0.001 | 1.283 | <0.001 | * |
| LI4 | Deqi vs BL | 6.23 | 3.76 | 4.83 | 7.64 | 9.087 | 29 | <0.001 | 1.659 | <0.001 | * |
| LI4 | Remove vs BL | 10.23 | 4.13 | 8.69 | 11.78 | 13.562 | 29 | <0.001 | 2.476 | <0.001 | * |
| LI4 | 15min vs BL | 11.03 | 4.87 | 9.22 | 12.85 | 12.416 | 29 | <0.001 | 2.267 | <0.001 | * |
| LI4 | 30min vs BL | 9.83 | 5.65 | 7.73 | 11.94 | 9.541 | 29 | <0.001 | 1.742 | <0.001 | * |

**Supplementary Table S4. Within-group Comparisons (vs. Baseline)**
