# Supplementary material for: Photoacoustic imaging of acupoint specificity and meridian transmission at Shousanli (LI10): a blood oxygen metabolism study
Source: Front Physiol. 2026 Aug 21;17:1911653. doi: 10.3389/fphys.2026.1911653 (PMC13541443; doi:10.3389/fphys.2026.1911653)
Supplement: Supplementary file 5 [file Table5.docx]

*Acu = Acupoint group. Non-mer = Non-meridian group. *: P < 0.0025.*

| Acupoint | Time | Acu ΔMean | Non-mer ΔMean | Diff | CI Low | CI High | t | df | Unadj.P | Cohen's d | Bonf.P | Sig. |
| --- | --- | --- | --- | --- | --- | --- | --- | --- | --- | --- | --- | --- |
| LI10 | Deqi | 5.60 | 0.53 | 5.07 | 3.44 | 6.69 | 6.311 | 40.2 | <0.001 | 1.630 | <0.001 | * |
| LI10 | Remove | 8.00 | 0.27 | 7.73 | 6.17 | 9.29 | 10.023 | 38.4 | <0.001 | 2.588 | <0.001 | * |
| LI10 | 15min | 8.23 | 0.37 | 7.87 | 5.90 | 9.83 | 8.131 | 33.2 | <0.001 | 2.099 | <0.001 | * |
| LI10 | 30min | 9.33 | -0.07 | 9.40 | 7.54 | 11.26 | 10.286 | 34.2 | <0.001 | 2.656 | <0.001 | * |
| LI11 | Deqi | 5.39 | 0.25 | 5.13 | 2.81 | 7.46 | 4.467 | 39.2 | 0.0001 | 1.153 | 0.0016 | * |
| LI11 | Remove | 6.62 | 0.00 | 6.62 | 4.85 | 8.38 | 7.518 | 49.4 | <0.001 | 1.941 | <0.001 | * |
| LI11 | 15min | 7.23 | 0.13 | 7.10 | 5.16 | 9.04 | 7.456 | 33.5 | <0.001 | 1.925 | <0.001 | * |
| LI11 | 30min | 7.37 | -0.03 | 7.40 | 5.58 | 9.22 | 8.250 | 35.1 | <0.001 | 2.130 | <0.001 | * |
| LI8 | Deqi | 3.95 | 0.48 | 3.47 | 1.36 | 5.58 | 3.338 | 34.9 | 0.0020 | 0.862 | 0.0320 | * |
| LI8 | Remove | 5.72 | -0.15 | 5.87 | 3.41 | 8.33 | 4.873 | 29.6 | <0.001 | 1.258 | <0.001 | * |
| LI8 | 15min | 5.18 | -0.19 | 5.37 | 2.69 | 8.05 | 4.075 | 33.5 | 0.0003 | 1.052 | 0.0048 | * |
| LI8 | 30min | 7.68 | -0.29 | 7.97 | 5.72 | 10.22 | 7.225 | 30.0 | <0.001 | 1.866 | <0.001 | * |
| LI4 | Deqi | 6.23 | -0.87 | 7.10 | 5.68 | 8.52 | 10.160 | 31.2 | <0.001 | 2.623 | <0.001 | * |
| LI4 | Remove | 10.23 | -0.23 | 10.47 | 8.90 | 12.03 | 13.634 | 31.0 | <0.001 | 3.520 | <0.001 | * |
| LI4 | 15min | 11.03 | -0.40 | 11.43 | 9.56 | 13.31 | 12.396 | 33.5 | <0.001 | 3.201 | <0.001 | * |
| LI4 | 30min | 9.83 | -0.80 | 10.63 | 8.51 | 12.75 | 10.246 | 29.8 | <0.001 | 2.646 | <0.001 | * |

**Supplementary Table S5. Between-group Comparisons of ΔSO₂**
