# Supplementary material for: Photoacoustic imaging of acupoint specificity and meridian transmission at Shousanli (LI10): a blood oxygen metabolism study
Source: Front Physiol. 2026 Aug 21;17:1911653. doi: 10.3389/fphys.2026.1911653 (PMC13541443; doi:10.3389/fphys.2026.1911653)
Supplement: Supplementary file 6 [file Table6.docx]

*Non-parametric alternative to repeated measures ANOVA.*

| Acupoint | Acu χ² | df | Acu P | Acu Kendall's W | Non-mer χ² | df | Non-mer P | Non-mer Kendall's W |
| --- | --- | --- | --- | --- | --- | --- | --- | --- |
| LI10 (Shousanli) | 57.213 | 4 | <0.001 | 0.477 | 17.991 | 4 | 0.0012 | 0.150 |
| LI11 (Quchi) | 49.542 | 4 | <0.001 | 0.413 | 20.239 | 4 | 0.0004 | 0.169 |
| LI8 (Xialian) | 41.033 | 4 | <0.001 | 0.342 | 24.638 | 4 | 0.0001 | 0.205 |
| LI4 (Hegu) | 75.865 | 4 | <0.001 | 0.632 | 43.991 | 4 | <0.001 | 0.367 |

**Supplementary Table S6. Friedman Test (Sensitivity Analysis)**
