# Supplementary material for: Photoacoustic imaging of acupoint specificity and meridian transmission at Shousanli (LI10): a blood oxygen metabolism study
Source: Front Physiol. 2026 Aug 21;17:1911653. doi: 10.3389/fphys.2026.1911653 (PMC13541443; doi:10.3389/fphys.2026.1911653)
Supplement: Supplementary file 7 [file Table7.docx]

*Non-parametric between-group comparison. r = effect size.*

| Acupoint | Time | Acu Median | Non-mer Median | U | Z | P | r |
| --- | --- | --- | --- | --- | --- | --- | --- |
| LI10 | Deqi | 5.0 | 1.0 | 803.5 | 5.226 | <0.001 | 0.675 |
| LI10 | Remove | 8.0 | 0.0 | 851.0 | 5.929 | <0.001 | 0.765 |
| LI10 | 15min | 7.0 | 0.0 | 858.0 | 6.032 | <0.001 | 0.779 |
| LI10 | 30min | 9.0 | 0.0 | 891.5 | 6.527 | <0.001 | 0.843 |
| LI11 | Deqi | 4.0 | 1.0 | 783.5 | 4.931 | <0.001 | 0.637 |
| LI11 | Remove | 6.0 | 0.0 | 832.0 | 5.648 | <0.001 | 0.729 |
| LI11 | 15min | 6.0 | 0.0 | 824.5 | 5.537 | <0.001 | 0.715 |
| LI11 | 30min | 7.0 | 0.0 | 859.0 | 6.047 | <0.001 | 0.781 |
| LI8 | Deqi | 5.0 | 1.0 | 672.0 | 3.282 | 0.0009 | 0.424 |
| LI8 | Remove | 7.0 | 0.0 | 780.0 | 4.879 | <0.001 | 0.630 |
| LI8 | 15min | 5.0 | 0.0 | 714.0 | 3.903 | 0.0001 | 0.504 |
| LI8 | 30min | 6.0 | 0.0 | 816.5 | 5.418 | <0.001 | 0.700 |
| LI4 | Deqi | 6.5 | -1.0 | 855.0 | 5.988 | <0.001 | 0.773 |
| LI4 | Remove | 11.0 | 0.0 | 885.5 | 6.439 | <0.001 | 0.831 |
| LI4 | 15min | 12.0 | 0.0 | 873.5 | 6.261 | <0.001 | 0.808 |
| LI4 | 30min | 10.0 | -1.0 | 854.0 | 5.973 | <0.001 | 0.771 |

**Supplementary Table S7. Mann-Whitney U Test (Sensitivity Analysis)**
